# Reveal the Heterogeneity in the Tumor Microenvironment of Pancreatic Cancer and Analyze the Differences in Prognosis and Immunotherapy Responses of Distinct Immune Subtypes

**DOI:** 10.3389/fonc.2022.832715

**Published:** 2022-02-17

**Authors:** Xiaoqin Wang, Lifang Li, Yang Yang, Linlin Fan, Ying Ma, Feifei Mao

**Affiliations:** ^1^ Department of Clinical Laboratory, The First Affiliated Hospital of Xi’an Jiaotong University, Xi’an, China; ^2^ Emergency Department, The First Affiliated Hospital of Xi’an Jiaotong University, Xi’an, China; ^3^ Department of Hepatobiliary and Pancreatic Surgery, The First People’s Hospital of Changzhou, Changzhou, China; ^4^ Tongji University Cancer Center, Shanghai Tenth People’s Hospital, School of Medicine, Tongji University, Shanghai, China

**Keywords:** pancreatic cancer, immune subtypes, heterogeneity, prognosis, microenvironment

## Abstract

**Purpose:**

The current clinical classification of pancreatic ductal adenocarcinoma (PDAC) cannot well predict the patient’s possible response to the treatment plan, nor can it predict the patient’s prognosis. We use the gene expression patterns of PDAC patients to reveal the heterogeneity of the tumor microenvironment of pancreatic cancer and analyze the differences in the prognosis and immunotherapy response of different immune subtypes.

**Methods:**

Firstly, use ICGC’s PACA-AU PDAC expression profile data, combined with the ssGSEA algorithm, to analyze the immune enrichment of the patient’s tumor microenvironment. Subsequently, the spectral clustering algorithm was used to extract different classifications, the PDAC cohort was divided into four subtypes, and the correlation between immune subtypes and clinical characteristics and survival prognosis was established. The patient’s risk index is obtained through the prognostic prediction model, and the correlation between the risk index and immune cells is prompted.

**Results:**

We can divide the PDAC cohort into four subtypes: immune cell and stromal cell enrichment (Immune-enrich-Stroma), non-immune enrichment but stromal cell enrichment (Non-immune-Stroma), immune-enriched Collective but non-matrix enrichment (Immune-enrich-non-Stroma) and non-immune enrichment and non-stromal cell enrichment (Non-immune-non-Stroma). The five-year survival rate of immune-enrich-Stroma and non-immune-Stroma of PACA-CA is quite different. TCGA-PAAD’s immune-enrich-Stroma and immune-enrich-non-Stroma groups have a large difference in productivity in one year. The results of the correlation analysis between the risk index and immune cells show that the patient’s disease risk is significantly related to epithelial cells, megakaryocyte-erythroid progenitor (MEP), and Th2 cells.

**Conclusion:**

The tumor gene expression characteristics of pancreatic cancer patients are related to immune response, leading to morphologically recognizable PDAC subtypes with prognostic/predictive significance.

## Introduction

Pancreatic ductal adenocarcinoma (PDAC) is one of the lethal malignant neoplasms around the world (1–4), and its genetic and phenotypic heterogeneity makes generally effective therapies ineffective (5–9). The salient feature of pancreatic cancer is that it has an immunosuppressive microenvironment, the prognosis of patients is poor, and most of the patients’ tumors will metastasize (10, 11). Research on the immune microenvironment of pancreatic cancer may help improve the therapeutic effect (12, 13). By detecting the expression of anti-tumor immune genes, markers that can predict patient response to treatment have been screened (14). In addition, mutations in genes such as PIK3CA, FGFR3, and TP53 have been shown to be related to tumor immune infiltration (15–18). Although we have a better understanding of the molecular mechanism and genetic background of pancreatic cancer, the 5-year survival rate for this disease is approximately 10% in the USA (19). Several phase III clinical trials that are effective for other cancers have not worked well in pancreatic cancer patients (7). Tumor heterogeneity and host differences will affect the characteristics of its tumor microenvironment. It is necessary to identify new biomarkers and explore new treatment approaches to provide more and more effective references for overcoming the immunosuppressive mechanism in the pancreatic cancer microenvironment.

The immune microenvironment plays an important role in tumor cell invasion and pancreatic cancer progression (20), and immune expression characteristics may affect the degree of inhibition of cancer cells. Invasive PDAC has epithelial-to-mesenchymal transition (EMT)-like characteristics and has been shown to be a poor prognostic factor for pancreatic cancer (21). The immune microenvironment with EMT-like tumors is conducive to tumor growth. Researchers reported on three subtypes of pancreatic cancer: classic, quasi-mesenchymal, and exocrine, and clarified the genetic markers of different subtypes, which may help to carry out more targeted treatments for patients (22). Other researchers have identified two tumor-specific subtypes based on gene expression: basal-like subtype and classical subtype (23). The classic subtype is consistent with the subtype described by Collisson et al. Tumor subtypes defined by exocrine-like genes have not been validated in its data set, and may be related to tissue contamination. Recently, researchers classified pancreatic cancer into four subtypes based on genomic studies—squamous cells, pancreatic progenitor cells, immunogenicity and abnormally differentiated endocrine and exocrine-identified the differences between pancreatic cancer subtypes and provided Different subtypes of treatment options (22, 24). Among them, squamous cells, pancreatic progenitor cells, and abnormally differentiated endocrine and exocrine (ADEX) subtypes correspond to the quasi-mesenchymal, classical, and exocrine-like subtypes reported by Collisson et al. (22). Recently, studies have shown that ADEX and immunogenic subtypes are related to the lower purity of the sample (24, 25). Although researchers have basically determined the characteristics of some pancreatic cancer subtypes, research conclusions about exocrine differentiation or immunogenic subtypes are still inconsistent.

Therefore, we aim to redefine the subtypes of PDAC and clarify its immune expression patterns, provide useful clues for exploring the different immunosuppressive mechanisms of PDAC, and use it in the stratification of patient clinical trials, so as to provide patients with PDAC more precise treatment.

## Results

### Classification of Distinct Tumor Microenvironment Subtypes

Single sample gene set enrichment analysis (ssGSEA) defines an enrichment score to indicate the absolute enrichment degree of the gene set in each sample in a given data set. The enrichment score of each immune category can be found in the R package GSVA In the realization (26). Firstly, ssGSEA algorithm (27) was utilized to analyze the expression profiling database of the PACA-AU pancreatic cancer in the International Cancer Genome Consortium (ICGC). We obtained the immune enrichment of the tumor microenvironment of each patient’s tumor tissue. And the tumor microenvironment-related genes come from the following references (**Table 1**).

**Table 1:** Immune-related gene signatures and their references.

Signature name	Reference
Immune enrichment score	Yoshihara et al. Nat Commun. 2013 (28)
6-gene IFN-γsignature	Chow et al. J Clin Oncol. 2016 (suppl) (29)
Activated stroma	Moffitt et al. Nat Genet. 2015 (30)
Immune cell subsets	Cancer Genome Atlas Network. Cell. 2015 (31)
T cells	Bindea et al. Immunity. 2013 (32)
CD8 Tcells	Bindea et al. Immunity. 2013 (32)
T. NK. metagene	Alistar et al. Genome Med. 2014 (33)
B-cell cluster	Iglesia et al. Clin Cancer Res. 2014 (34)
Macrophages	Bindea et al. Immunity. 2013 (32)
Cytotoxic cells	Bindea et al. Immunity. 2013 (32)
Immunophenoscore	Charoentong et al. Cell Rep. 2017 (35)
T cell-inflamed GEP	Cristescu et al. Science. 2018 (36)
Expanded immune signature	Ayers et al. J Clin Invest. 2017 (37)
TGF-β-associated ECM	Chakravarthy et al. Nat Commun. 2018) (38)
MDSC	Yaddanapudi et al. Cancer Immunol Res. 2016 (39)
CAF	Calon et al. Cancer Cell. 2012 (40)
TAM M2/M1	Beyer et al. PLoS One. 2012 (41)
CD8 T cell exhaustion	Giordano et al. EMBO J. 2015 (42)
T cell exhaustion early/late stage	Philip et al. Nature. 2017 (43)
Nivolumab responsive	Riaz et al. Cell. 2017 (44)

**Figure 1:**
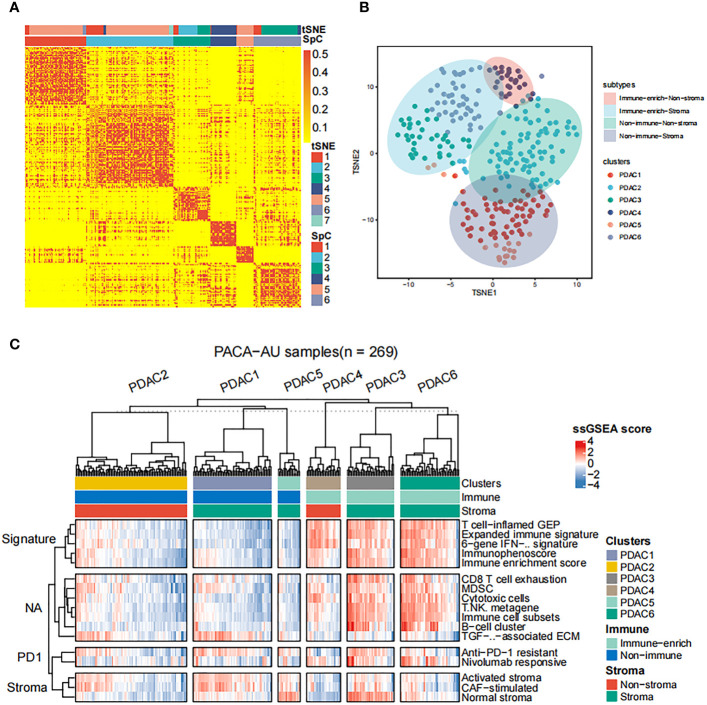
Classification of distinct tumor microenvironment subtypes **(A)** Spectral classification of tumor microenvironment in PACA-AU alignment. This plot shows a heat map of the ssGSEA score, estimated using the gene set from the ICGC database. Based on tSNE cluster analysis, 7 subgroups were obtained, namely PDAC1, PDAC2, PDAC3, PDAC4, PDAC5, PDAC6, PDAC7. Based on Spectral classification, 6 subgroups were obtained, namely PDAC1, PDAC2, PDAC3, PDAC4, PDAC5, PDAC6. **(B)** tSNE classification of tumor microenvironment in PACA-AU cohort. **(C)** This figure shows the 4 immune subtypes of the PACA-AU cohort based on ssGSEA analysis and the main signatures of each subtype.

Subsequently, we apply the spectral clustering algorithm to extract different categories based on the ssGSEA scores (**Figure 1A**). Meanwhile, we used t-distributed stochastic neighbor embedding (tSNE) to show the groups (**Figure 1B**), and revealed an immune-enriched subtype (Immune-enrich) exists in the cohort, and the rest are of the Non-immune type, that is, less immune infiltration (**Figure 1C**). In addition, even in the presence of a large population of immune cells, stromal cells also play vital roles in tumor immunity evasion. Therefore, we further dissected the enrichment of stromal cells in the patient’s gene expression profile. Also using ssGSEA analysis, we found that the cohort had characteristics of activated stromal response (**Figure 1C**). Based on the above classification, we can divide the pancreatic cancer cohort into four subtypes: immune cell and stromal cell enrichment (Immune-enrich-Stroma), non-immune enrichment but stromal cell enrichment (Non-immune-Stroma), Immune enrichment but non-matrix enrichment (Immune-enrich-non-Stroma) and non-immune enrichment and non-stromal cell enrichment (Non-immune-non-Stroma). Immune-enrich-Stroma subtypes mainly enrich tumor immune-related molecular signatures, including T cell-inflamed GEP, Expanded immune signature, Immunophenoscore, Immune enrichment score, CD8 T cell exhaustion, myeloid-derived suppressor cells (MDSC), cytotoxic cells, Immune cell subset, etc. At the same time, it also enriches PD1 and stroma related signatures, including anti-PD-1 resistant, nivolumab responsive and normal stroma. The signatures of Non-immune-Stroma subtypes mainly include anti-PD-1 resistant, activated stroma, CAF-stimulated, and normal stroma, while its immune-related family features are very low. Immune-enrich-non-Stroma subtypes mainly enrich tumor immune-related signatures, including T cell-inflamed GEP, Expanded immune signature and cytotoxic cells, etc., while its stromal signatures expression is very low. Non-immune-non-Stroma subtypes, as the name suggests, are rarely enriched in tumor immunity and stromal signatures.

### Comparison of the Striking Differences in the Immune Microenvironment of the Four Subtypes

**Figure 2:**
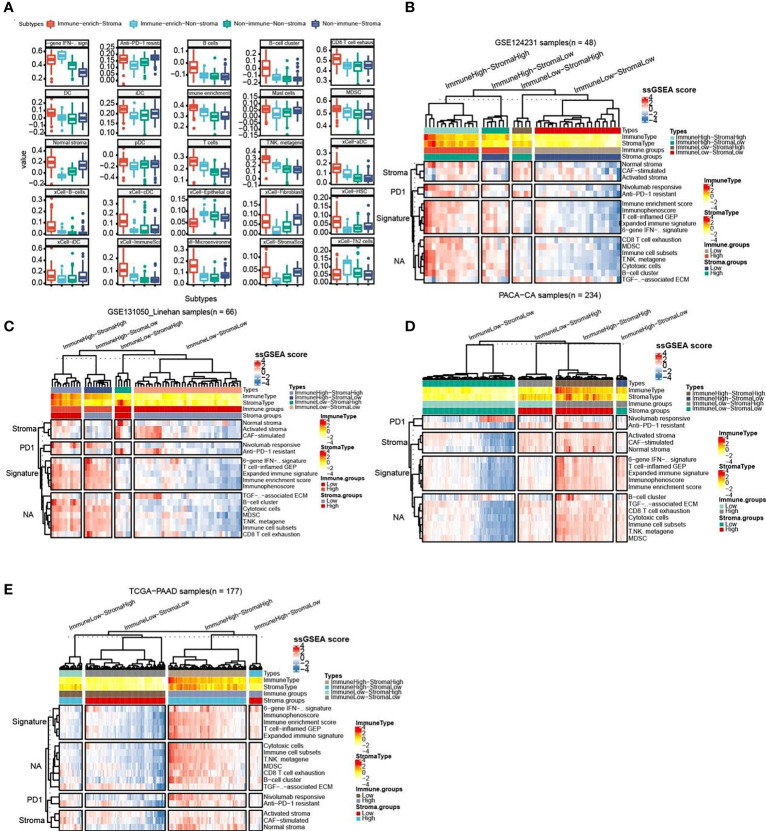
Comparison of the striking differences in the immune microenvironment of the four subtypes. **(A)** Comparison of the striking differences in the immune microenvironment of the four subtypes. Red represents immune-enrich-stroma subtype, Light_blue represents immune-enrich-non-stroma subtype, Green represents non-immune-non-stroma subtype, and Navy blue represents non-immune-stroma subtype. **(B)** Immune-enrich-Stroma, Immune-enrich-non-Stroma, Non-immune-Stroma and Non-immune-non-Stroma types in the GSE124231 data set (n=48). **(C)** Four types in the GSE131050 data set (n=66). **(D)** Four types in the PACA-CA data set (n=234). **(E)** Four types in the TCGA-PAAD database (n = 177).

As follow, the four subtypes have the following immune differences (**Figure 2A**). Patients with immuno-enriched subtypes (**Figure 2A** red and light blue boxes) showed significant enrichment in the characteristics of recognizing immune cells or immune responses (all P <0.05). We further compared the difference in gene expression between immune-enriched and non-immune-enriched patients, mainly using the limma algorithm, and P<0.05 as the standard of significant difference (**Table S1**). At the same time, the significantly different genes of stromal cell enrichment and non-stromal enrichment was compared (**Table S2**).In order to verify the accuracy and consistency of the analysis method, we use the same strategy to predict the enrichment of other data. The first 50 genes that are differentially up-regulated are selected to construct a gene set, and the ssGSEA algorithm is used to predict the enrichment of other data. In addition, select significantly different immune activity or immune cell-related genes to verify their enrichment. The analysis results show that the GSE124231 data set (**Figure 2B**, n=48), the GSE131050 data set (**Figure 2C**, n=66), the PACA-CA data set (**Figure 2D**, n=234) and the TCGA-PAAD database (**Figure 2E**, n = 177) can be divided into immune enrichment and stromal cell enrichment groups. According to the constructed gene set, samples of different data sets can be divided into immune-enrich-Stroma, immune-enrich-non-stroma, non-immune-stroma and non-immune-non-stroma types. And immune enrichment type samples are mainly enriched for immune-related signatures, such as immune enrichment score, immunophenoscore, Immune cell subsets, etc. Stromal cell enrichment types mainly enrich stroma-related signatures, such as normal stroma, activated stromanivolumab responsive, etc. The above results show that the accuracy and consistency of our classification and research methods are trustworthy.

### Four Immune Subtypes Are Related to Clinical Characteristics and Survival Prognosis

Based on the previous results, we have divided patients into 4 different subtypes of immune enrichment and stromal cell enrichment. Therefore, we need to further compare the clinical characteristics of different types and try to explore the relationship between each type and patient survival prognosis. Firstly, we sequentially compared the clinical information between different subtypes in the PACA-AU, PACA-CA and TCGA-PAAD cohorts. Statistics showed that there were significant differences among subtypes in the PACA-AU cohort, which included donor_sex, donor_vital_status, donor_relapse_type, donor_age_at_diagnosis and enrollment, donor_survival_time, donor_interval_up, donor_interval_up, donor_interval_up (**Table S3**). In the PACA-CAcohort, clinical markers such as donor_age_at_diagnosis and enrollment, donor_age_at_last_followup, donor_survival_time, donor_interval_of_last_followup are significantly different among subgroups (**Table S4**). Age_at_initial_pathologic_diagnosis, family_history_of_cancer (%), history_of_chronic_pancreatitis (%), history_of_diabetes (%) and other clinical indicators were significantly different among 4 subsets in the TCGA-PAAD cohort (**Table S5**).

**Figure 3:**
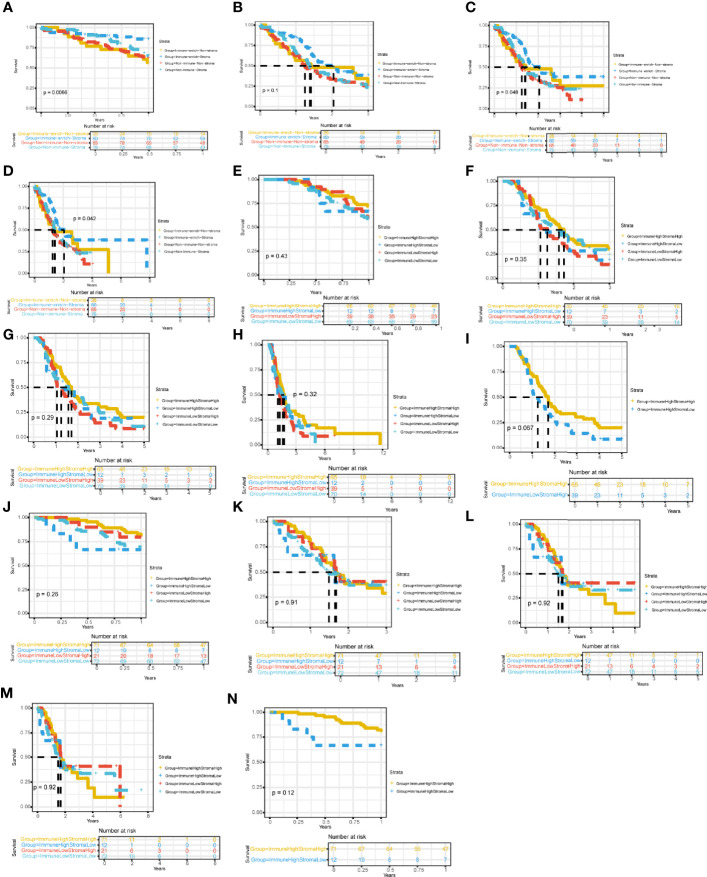
Four immune subtypes are related to clinical characteristics and survival prognosis Comparison of survival rates between subgroups in different cohorts **(A)** Comparison of 1-year survival rate of PACA-AU cohort. **(B)** Comparison of 3-year survival rate of PACA-AU cohort. **(C)** Comparison of 5-year survival rate of PACA-AU cohort. **(D)** Comparison of survival rates of all PACA-AU cohort. **(E)** Comparison of 1-year survival rate of PACA-CA cohort. **(F)** Comparison of 3-year survival rate of PACA-CA cohort. **(G)** Comparison of 5-year survival rate of PACA-CA cohort. **(H)** Comparison of survival rates of all PACA-CA cohort. **(I)** Comparison of survival rates of the Immune-enrich-Stroma and Non-immune-Stromasubtypes. **(J)** Comparison of 1-year survival rate of TCGA-PAAD cohort. **(K)** Comparison of 3-year survival rate of TCGA-PAAD cohort. **(L)** Comparison of 5-year survival rate of TCGA-PAAD cohort. **(M)** Comparison of survival rates of all TCGA-PAAD cohort. **(N)** Comparison of survival rates of the Immune-enrich-Stroma and Immune-enrich-non-Stroma subtypes.

Then, we successively explored the relationship between different subgroups in the cohort and the survival prognosis of patients. In the PACA-AU cohort, the 1-year (**Figure 3A**) and 5-year (**Figure 3C**) survival rates between different subtypes are significantly different (p.value <0.05), and the survival rate of the Immune_enrich_Stroma subgroup is higher than that of the other three groups. However, the difference in 3-year survival rates between patient groups was not significant (**Figure 3B**). Finally, we compared the survival rates of all PACA-AU patients (8 years) and found that the survival rates of different subgroups are still significantly different (p.value <0.05) (**Figure 3D**). Similarly, we compared the survival rates of patients in the PACA-CA cohort for 1 year (**Figure 3E**), 3 years (**Figure 3F**), 5 years (**Figure 3G**) and all patients (12 years) (**Figure 3H**) in detail, and found There are no significant differences between different subgroups. It is worth mentioning that there is a relatively large difference in the five-year survival rate between the immune-enrich-stroma and non-immune-stroma groups of PACA-CA (**Figure 3I**). The analysis results of the TCGA-PAAD cohort showed that the survival rates of patients in different subgroups were 1 year (**Figure 3J**), 3 years (**Figure 3K**), 5 years (**Figure 3L**) and all patients (8 years) (**Figure 3M**). It was found that there were no significant differences between the different subgroups. However, the one-year survival rate difference between immune-enrich-stroma and immune-enrich-non-stroma groups is relatively large (**Figure 3N**). In general, the classification of the PACA-AU cohort can provide an important reference for their clinical survival prognosis.

### Prognostic Prediction Model Based on Signatures of Tumor Microenvironment

**Figure 4:**
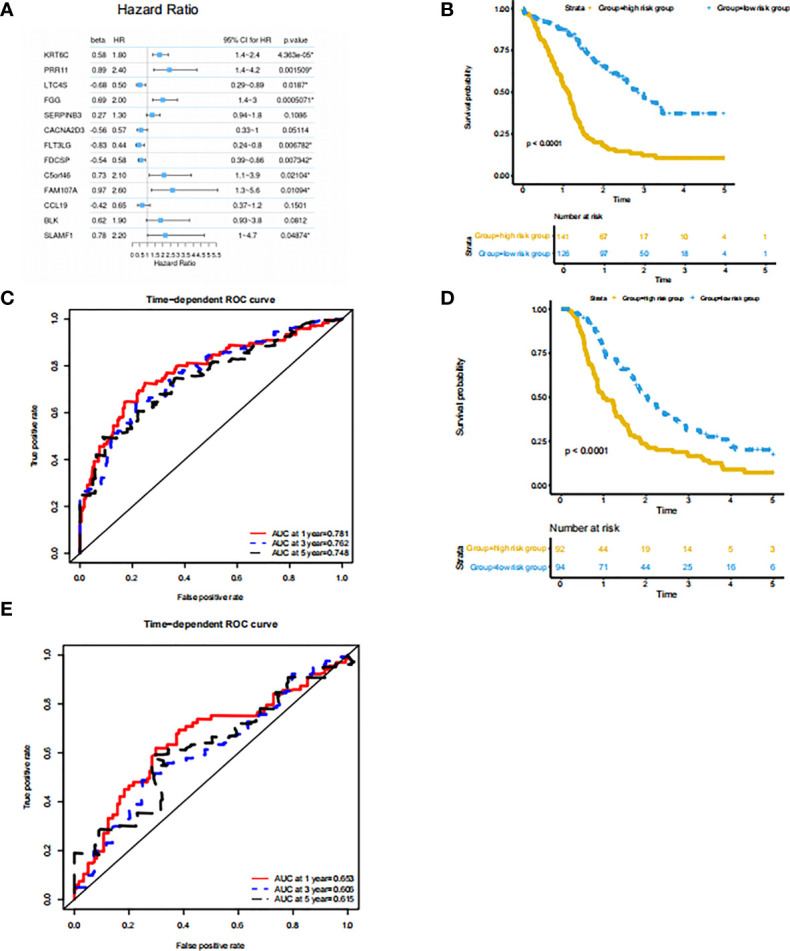
Prognostic prediction model based on signatures of tumor microenvironment **(A)** features: significant factor name; multi_beta: Cox multiple regression coefficient; multi_HR: Cox multiple regression risk ratio; multi 95% CI for HR: Cox multiple regression risk ratio 95% confidence interval; Forest diagram: horizontal line shows the confidence interval interval, and the dot represents the hazard ratio; multi_p.value: Cox multiple regression proportional hazard hypothesis test P value. **(B)** Survival curve of the high and low risk groups in the training set. The horizontal axis represents time (unit: day), the vertical axis represents survival rate. A flat curve represents a high survival rate or a longer survival period, and a steep curve represents a low survival rate or a shorter survival period. **(C)** ROC curve of the training set prediction model. The horizontal axis is the false positive rate FP, and the vertical axis is the true positive rate TP. The legend in the upper left corner corresponds to the AUC value of the ROC curve for different survival periods. **(D)** Survival curves of the high- and low-risk groups in the PACA-CA testing set. **(E)** ROC curve of PACA-CA test set prediction model. The horizontal axis is the false positive rate FP, and the vertical axis is the true positive rate TP. The legend in the upper left corner corresponds to the AUC value of the ROC curve for different survival periods. The horizontal axis is the false positive rate (FP), and the vertical axis is the true positive rate (TP). The legend in the upper left corner corresponds to the AUC value of the ROC curve for different survival periods.

Since the subtype classification in the PACA-AU cohort has a strong correlation with survival prognosis, we use PACA-AU data as training data, and PACA-CA and TCGA-PAAD as test data to construct a prognostic prediction model. Firstly, PACA-AU data is treated as training data for parameter training of prediction models and selection of related gene sets. PACA-CA and TCGA-PAAD are regarded as testing data to test the parameters given by the training set and the predictive ability of the gene set. Then, use the cox regression algorithm to initially screen the genes that are significantly related to the patient’s overall survival (P<0.05), and use the LASSO algorithm to further screen these genes. In the end, the best gene panel is obtained, and the forest diagram of the multivariate COX regression model is drawn (**Figure 4A**). In detail, those genes are KRT6C, PRR11, LTC4S, FGG, SERPINB3, CACNA2D3, FLT3LG, FDCSP, C5ORF46, FAM107A, CCL19, BLK, SLAMF1 and their multiple regression coefficients are 0.58, 0.89, -0.68, 0.69, 0.27, -0.56, -0.83, -0.54, 0.73, 0.97, -0.42, 0.62, 0.78. Subsequently, based on the expression level and multiple regression coefficients of gene panel obtained above, calculate their risk score. We further divided patients into high-risk groups and low-risk groups based on the risk index of the sample. Kaplan-Meier survival analysis was performed and showed in survival curve. There is a significant difference in survival probability between the high-risk group and the low-risk group in PACA-AU cohort (p <0.05) (**Figure 4B**). At the same time, we drew the ROC curve of the one-year, three-year, and five-year survival period of the patients in the training set based on the risk index (**Figure 4C**). However, there was no significant difference in the survival probability between the high-risk group and the low-risk group in the TCGA-PAAD testing set.

At the same time, we drew the ROC curve of patient survival in PACA-CA (**Figure 4E**) cohorts based on the risk index. The ROC curve of the prediction model of the PACA-CA training set shows that the prediction model is relatively ideal, and the prediction of the 1-year survival period is slightly better than the 3-year and 5-year survival periods. In addition, the prediction effect of the PACA-CA testing set (**Figure 4D**) is slightly inferior to that of the PACA-AU training set, except for the 5-year survival period of the TCGA-PAAD testing set. Overall, the prognosis prediction model can better predict the grouping of patients based on the risk index, which provides guidance for the prognosis prediction of patients.

### Immune Cells Related to Risk Index

**Figure 5:**
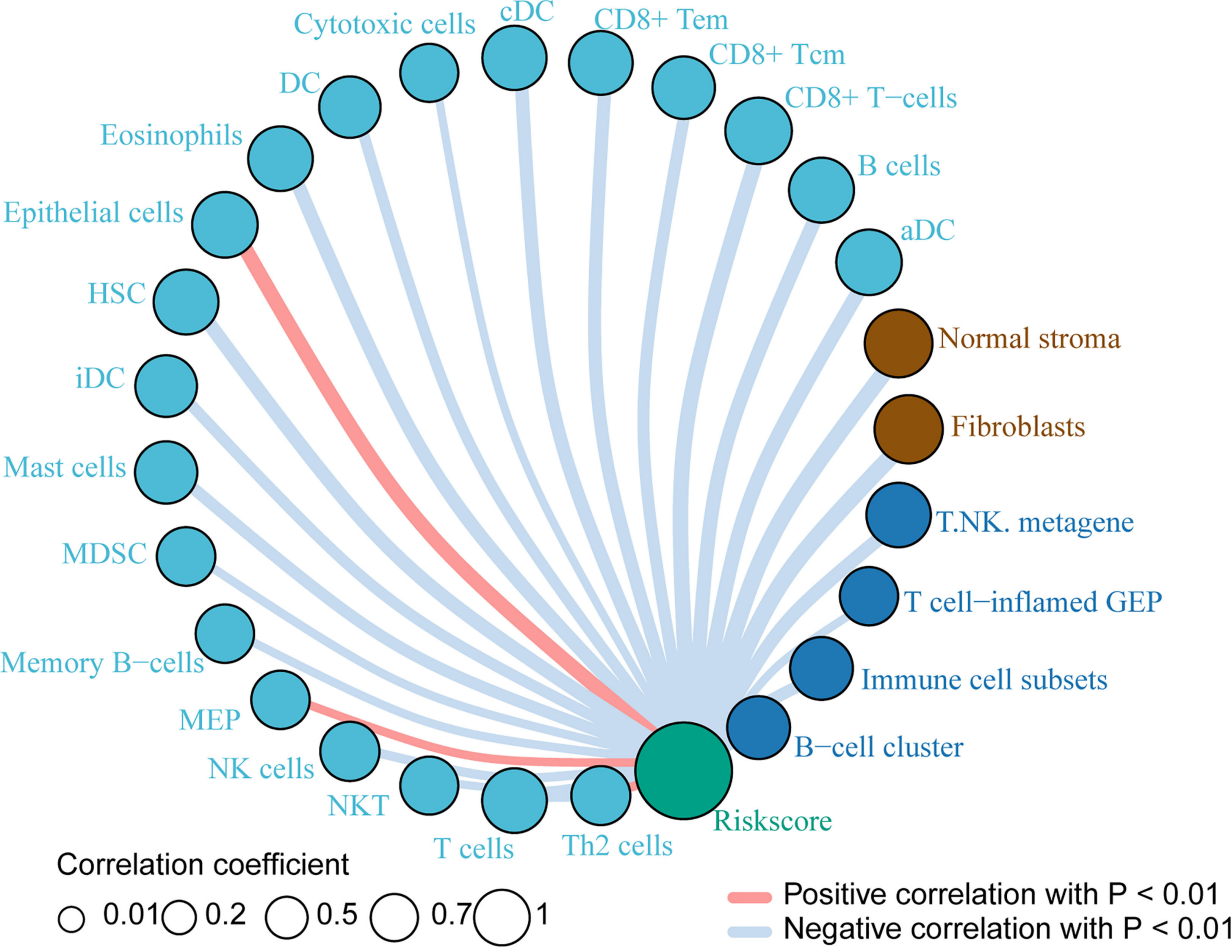
Immune cells related to risk index Immune cells associated with the risk index of PACA-AU patients. The red line indicates a positive correlation between the risk index and immune cells, and the gray line indicates a negative correlation between the risk index and immune cells. The size of the circle indicates different correlation coefficients, and the larger the area of the circle, the larger the correlation coefficient.

Based on the previous results, we want to know which immune cells are specifically related to the risk index of the PACA-AU cohort. Therefore, we used the sample risk index to make further correlation analysis with the expression of various immune cells and immune molecules. The results showed that the patient’s risk index and epithelial cells, megakaryocyte-erythroid progenitor (MEP), and Th2 cells showed a positive correlation with p<0.01. In addition, T cells, NK cells, memory B-cells, mast cells and other immune cells have a negative correlation with p <0.01 (**Figure 5**).

## Discussion

In this study, we used the ssGSEA algorithm to calculate the ssGSEA scores of PACA-AU pancreatic cancer patients, and then combined the Spectral clustering algorithm to extract the 4 subtypes in the cohort. We further compared the differences in the immune microenvironment of the four subtypes, and screened the immune enrichment and stromal enrichment molecular markers. Genes with significant differences are mostly related to immunity in (**Table S1**). For example, changes in the expression of PTPRCAP affect the survival rate of cancer patients (45), and the single nucleotide polymorphism (SNP) of PTPRCAP is associated with the susceptibility of gastric cancer (46). Natural killer cell granule protein 7 (NKG7) is related to inflammatory diseases (47), and its lack will result in a significant reduction in IFN-γ produced by T cells and NK cells. In addition, NKG7 is related to the cytotoxic degranulation of CD8+ T cells (48). Researchers have discovered that CD96 can serve as a new immune checkpoint receptor target for T cells and natural killer cells (49). Similarly, we observed the top genes and their stromal functions in (**Table S2**). For example, Slits3 is expressed in primary bone marrow stromal and bone marrow-derived endothelial cells and stromal cell lines, and plays a role in *in vitro* migration and *in vivo* homing of hematopoietic stem and progenitor cells (50). SPARC is a stromal cell protein, which can be produced by cells associated with tumor stromal cells and has high expression levels in many cancers. It plays an important role in the fibroproliferative reaction of tumors (51).

Using the same research method, it was verified in the GSE124231 (n=48), GSE131050_Linahan (n=66), PACA-CA (n=234), TCGA-PAAD (n=177) cohorts, and the typing was accurate in different cohorts. Further compare the clinical information of patients in the cohort, and in-depth exploration of the difference in survival of patients with different subgroups. We found that in the PACA-AU cohort, the 1-year, 5-year, and 8-year survival times of different subsets patients were significantly correlated. Next, cox regression combined with Lasso algorithm was performed to construct a multivariate COX model. Calculate the patient’s risk index based on gene expression level and multiple regression coefficients, and divide the patients into high-risk groups and low-risk groups based on the risk index. Interestingly, the PACA_AU and PACA-CA risk indexes are significantly correlated with the survival level of patients.

In PADA-AC and TCGA-PAAD, the survival time difference between different immune subgroups is not significant. Only the five-year survival of immune-enrich-stroma and non-immune-stroma group in PACA-CA cohort and the one-year survival of immune-enrich-Stroma and immune-enrich-non-Stroma group in TCGA-PAAD cohort are relatively large. On the one hand, the cohort clustering algorithm may not cover all patients in the cohort, on the other hand, it may also be because the cohort samples are not large enough, and the representativeness of the statistical results needs to be further improved.

We initially explored the types of immune cells related to the risk index, and we identified immune cells that are positively and negatively related to the risk index. This research lays the foundation for the subsequent in-depth exploration of the correlation mechanism between immune cells and patient disease risk. However, only analyzing the types of immune cells is insufficient for the study of the mechanism. In the later stage, we will conduct more in-depth analysis and verification of important immune cells and their molecular signatures.

## Methods

### Project and Sample

Dataset of 461 PACA-AU donors were downloaded from ICGC database (https://dcc.icgc.org/projects/PACA-AU) with detailed clinical information. The independent datasets used for verification come from GSE124231, GSE131050_Linehan, PACA-CA and TCGA-PAAD projects, including 48, 66, 234 and 177 donors respectively. Moreover, patients in the PACA-CA and TCGA-PAAD cohorts had detailed clinical information.

### Bioinformatics Analysis

1) ssGSEA algorithm: Use the R package “GSVA” and use ssGSEA to explore the PACA-AU pancreatic cancer expression profile data of the ICGC database, and analyze the immune enrichment of each patient’s tumor microenvironment. Additionally, the gene expression of all samples were took as the input and ssGSEA algorithms were occupied to determine the proportion of the various immune cells of all PDAC samples. The immune gene signatures were listed in the **Table 1**. According to the immune enrichment status of PACA-AU samples, they are divided into immune cells and stromal cell enriched (immune-enrich-stroma), non-immune enrichment but stromal cell enrichment (non-immune-stroma), and immune-enriched but Non-matrix enrichment (immune-enrich-non-stroma) and non-immune enrichment and non-stromal cell enrichment (non-immune-non-stroma). According to the ssGSEA score obtained by each sample, the Spectral clustering algorithm is used to extract different classifications. In addition, the R package “limma” was used to analyze immuno-enriched and non-immune-enriched patients, as well as the significantly different genes of stromal cell enrichment and non-matrix enrichment, and P<0.05 was taken as the significant difference.

2) The unsupervised clustering of the data set was performed mainly based on tSNE which embedded in t-distributed random neighborhoods (45). In this study, we use tSNE to show the different subgroups of the PACA-AU cohort.

3) We performed Kaplan-Meier survival analysis on the samples and plotted survival curves. Survival analysis divided the samples into high-index groups and low-index groups based on the median. Data visualization is mainly done in the R environment (version 4.1.0). Kaplan-Meier survival analysis relies on the use of the “survival” package. The ROC curve is drawn based on the’survivalROC’ package.

4) Prognosis prediction model establishment process: a). Use the training set to perform unit cox regression on each gene to initially screen disease-related genes; b). After obtaining all cox significant genes in all units, perform 1000X LASSO regression to calculate the frequency of each gene and rank it; c). According to the sorting result of the previous step, build the gene set incrementally. Use each gene set to perform multiple cox regression to get the contribution of each gene; d). Obtain the optimal gene set according to the gene contribution degree, and perform multiple cox regression analysis on these genes. Finally, we determined the regression coefficient of each gene; e). Calculate the death risk score of each patient through regression coefficients; f). The death risk score model is tested in the training set (comparing the predicted situation with the actual situation); g). The same model is tested in the independent testing set at the beginning (comparison of the predicted situation with the actual situation).

5) Construct the optimal multivariate COX model based on the Lasso algorithm. This analysis uses the LASSO algorithm for gene screening: In the field of statistics and machine learning, Lasso algorithm (least absolute shrinkage and selection operator, also translated as minimum absolute shrinkage and selection operator, lasso algorithm) is a regression analysis method that simultaneously performs feature selection and regularization (mathematics).It aims to enhance the predictive accuracy and interpretability of statistical models. Lasso adopts the linear regression method of L1-regularization, so that the weight of some learned features is 0, so as to achieve the purpose of sparseness, selection of variables, and construction of the best model. The characteristic of LASSO regression is to perform variable selection and regularization while fitting a generalized linear model. Therefore, regardless of whether the target dependent variable (dependent/response variable) is continuous, binary or discrete, it can be modeled by LASSO regression and then predicted.

6) We use the Lasso algorithm (glmnet package) to select the best gene model based on the COX multiple regression model, and finally draw the unit cox regression model forest diagram based on the gene Panel as follows: We calculate the risk score (Risk Score) of each patient based on the expression of the gene Panel and the multiple regression coefficient. The formula is as follows:


$$\text{Riskscore}=\sum_{\text{i}=1}^{\text{n}}\beta \text{i}*\text{xi}$$

xi represents the expression level of each gene in the Panel, βi is the multivariate COX regression beta value (multi_beta) corresponding to each gene.

## Data Availability Statement

The original contributions presented in the study are included in the article/**Supplementary Material**. Further inquiries can be directed to the corresponding author.

## Author Contributions

FM and XW conceived this project. XW, LL, LF, and YM collected the data. YY and FM analyzed and interpreted the data. XW and FM performed the statistical analyses and wrote the manuscript. All authors have reviewed the manuscript and approved the final version.

## Funding

This work was financially supported by National Natural Science Foundation of China (82002480).

## Conflict of Interest

The authors declare that the research was conducted in the absence of any commercial or financial relationships that could be construed as a potential conflict of interest.

The handling editor declared a shared parent affiliation with several of the authors LL, LF, YM, and XW at time of review.

## Publisher’s Note

All claims expressed in this article are solely those of the authors and do not necessarily represent those of their affiliated organizations, or those of the publisher, the editors and the reviewers. Any product that may be evaluated in this article, or claim that may be made by its manufacturer, is not guaranteed or endorsed by the publisher.
